# AI‐Driven Revolution of Medical Robotics Across Surgical Innovation, Rehabilitation Intelligence, and Multimodal Healthcare Delivery

**DOI:** 10.1002/mco2.70597

**Published:** 2026-03-08

**Authors:** Fanxuan Chen, Haoman Chen, Tao Yu, Ruoyun Wang, Yi Wang, Xian Zhang, Jiachen Li, Kaishuo Liu, Darong Hai, Xueying Bao, Zefei Mo, Dongren Yang, Zhao Wang, Youhui Lin, Qinghua Xia, Gen Yang, Jianwei Shuai

**Affiliations:** ^1^ Oujiang Laboratory (Zhejiang Lab for Regenerative Medicine, Vision, and Brain Health), Wenzhou Institute University of Chinese Academy of Sciences Wenzhou China; ^2^ School of Biomedical Engineering, School of Ophthalmology and Optometry Eye Hospital Wenzhou Medical University Wenzhou China; ^3^ The First School of Medicine, School of Information and Engineering Wenzhou Medical University Wenzhou China; ^4^ The Second Clinical Medical College of Wenzhou Medical University Wenzhou China; ^5^ School of Stomatology Wenzhou Medical University Wenzhou China; ^6^ School of Nursing Wenzhou Medical University Wenzhou China; ^7^ National Institute for Data Science in Health and Medicine Xiamen University Xiamen China; ^8^ Department of Physics, Research Institute for Biomimetics and Soft Matter, Fujian Provincial Key Laboratory for Soft Functional Materials Research Xiamen University Xiamen China; ^9^ Ningbo Innovation Centre Zhejiang University Ningbo China; ^10^ State Key Laboratory of Nuclear Physics and Technology, School of Physics Peking University Beijing China; ^11^ Shenzhen Hospital, Medical Cancer Center the University of Hong Kong Shenzhen China

**Keywords:** artificial intelligence, medical robotics, rehabilitation support, surgical assistance

## Abstract

Artificial intelligence (AI) is catalyzing a paradigm shift in medical robotics, transforming medical robots from teleoperated tools into intelligent partners across clinical domains. This evolution is pivotal in addressing global challenges like aging populations, driven by core AI pillars—including computer vision (CV), deep reinforcement learning, and large language models (LLMs)—that support perception, decision‐making, and naturalistic communication, enabling varying degrees of autonomy and adaptive care. However, the literature still lacks a holistic analysis that integrates these advances and tackles the translational challenges hindering clinical adoption. This review bridges this gap by systematically charting the evolution of AI‐driven robotics across intelligent surgery, adaptive rehabilitation, and multimodal healthcare delivery. We dissect the core technologies powering this revolution, from digital twins for surgical simulation to LLMs for enhanced human–robot interaction, and critically analyze the associated technical, ethical, and regulatory hurdles. By synthesizing current progress and outlining future frontiers, including embodied AI, nanorobotics, and the concept of the AI‐augmented surgeon, this review provides a comprehensive roadmap for accelerating the translation of intelligent medical robotics into routine clinical practice.

## Graphical abstract

The figure illustrates the background, applications, limitations, and future trends of medical robotics in the context of global aging and strained medical resources. It highlights issues such as the increasing demand for healthcare due to aging populations, with a focus on applications in surgery, rehabilitation, medical examination, and assistance. Key challenges include high costs, lack of standardization, and human–machine trust deficits. The future trends section emphasizes the role of autonomous systems in decision‐making, machine perception, and the integration of safety measures like algorithmic registration and new materials for improved security and efficiency in medical robotics.

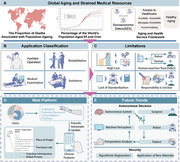

## 1 Introduction

The field of medical robotics is undergoing a profound transformation, evolving from sophisticated tools for teleoperation into intelligent partners in clinical practice. Historically, the paradigm was dominated by master–slave systems, exemplified by surgical platforms like the da Vinci Surgical System. These systems excel at extending a surgeon's dexterity and providing enhanced visualization, effectively translating human commands into precise mechanical actions. While revolutionary, this model represents an extension of human capability rather than a fundamental shift in medical workflow. However, the escalating pressures of an aging global population and the uneven distribution of medical resources—with a projected shortfall of 10 million health workers by 2030 [1]—demand solutions that offer not just precision, but also scalability, autonomy, and efficiency. The domain of medical robotics stands as a pivotal driver of 21st‐century technological advancement, undergoing significant expansion in both industrial scale and financial investment. According to a market analysis report, the global medical robotic systems market was valued at $2.26 billion in 2018 and is projected to reach $10.71 billion by 2026, exhibiting a remarkable compound annual growth rate of 21.5% during the forecast period [2]. As a core component of smart healthcare, medical robotics is poised to play an increasingly important role in the future global healthcare landscape.

At the heart of this paradigm shift lies artificial intelligence (AI), the engine powering the transition from teleoperated systems to intelligent robotics [3]. This “AI‐driven revolution” is not a monolithic development but a convergence of technologies that grant robots the ability to perceive, reason, and act with increasing levels of autonomy. Key AI pillars—including machine learning for diagnostic pattern recognition, computer vision (CV) for perception and scene understanding, and natural language processing (NLP) for seamless human–robot interaction—are redefining the boundaries of what medical robots can achieve. These technologies enable robots to process vast streams of multimodal data in real‐time, adapt to dynamic clinical environments, and assist in complex decision‐making. This revolution fundamentally alters the human–machine relationship, moving towards a synergistic collaboration where the robot can support clinicians by predicting likely next steps, optimizing procedures, and provididing data‐driven insights. It is a departure from pre‐programmed automation, heralding an era of adaptive and context‐aware robotic assistance in surgery, rehabilitation, and patient care.

Despite the rapid technological advancements, recent literature often fails to provide a holistic and forward‐looking perspective. Existing reviews tend to be compartmentalized, focusing on isolated domains such as the mechanical performance of surgical systems while overlooking economic feasibility, ethical implications, or cross‐platform interoperability [4]. Furthermore, many analyses lag behind the accelerating convergence of technology, notably overlooking the integration of large language models (LLMs) and heterogeneous computing architectures that are reshaping robotic control and data processing [5, 6]. A critical gap persists in creating a unified framework that addresses the interdisciplinary and translational challenges hindering widespread clinical adoption.

Accordingly, the primary objective of this review is to bridge these gaps by providing a comprehensive, integrated analysis of the AI‐driven revolution in medical robotics. Specifically, this review will first systematically chart the evolution from tele‐manipulation to intelligent systems across key domains, including surgery, rehabilitation, diagnostics, and hospital services. It will then critically analyze the core AI technologies driving this transformation and explore the technical, ethical, and regulatory challenges they introduce, such as algorithmic bias, data privacy, and accountability in semiautonomous and autonomous decision‐making. Furthermore, this review will propose a unified framework for evaluating and implementing intelligent medical robots, addressing crucial translational factors like cost‐effectiveness, human–computer interaction (HCI), workforce training, and standardization. Finally, looking toward the future, this review will discuss the conceptual architecture for a resource integration platform designed to consolidate information and facilitate a more cohesive development ecosystem for the next generation of medical robots.

## 2 Core AI Pillars Powering Next‐Generation Medical Robots

Medical robots are intelligent systems engineered for clinical applications, distinguished by their capacity for autonomous decision‐making—the ability to perceive their environment and execute tasks with reduced reliance on continuous human control [7, 8]. The AI‐driven revolution in medical robotics is not the result of a single technological breakthrough but is constructed upon a set of foundational, synergistic AI pillars. These technologies endow robots with a spectrum of cognitive capabilities—from perception and decision‐making to interaction and simulation—transforming them from passive, pre‐programmed tools into intelligent, adaptive partners in the clinical workflow. This chapter dissects the four core AI pillars that underpin this transformation, clarifying how they fundamentally reshape the capabilities of medical robots and drive emerging applications across clinical domains [9].

### 2.1 Machine Learning and CV for Perception and Environmental Understanding

If sensors are the “eyes” of a medical robot, then machine learning, and specifically CV, serves as its “visual cortex” and computational “brain.” This technological pillar addresses the most fundamental challenge for any robot operating in a clinical setting: reliably perceiving and interpreting a complex, dynamic, and high‐stakes environment [10]. Traditional robotic vision systems merely provided magnified or 3D images, acting as a simple extension of the surgeon's own sight. In contrast, an AI‐driven CV represents a paradigm shift from “passive presentation” to “active understanding.” By training deep learning models on vast datasets of medical imagery, robots can now perform a host of revolutionary tasks in real‐time [11]. This active understanding manifests in several critical capabilities. First, real‐time anatomical recognition and segmentation can automatically delineate organs, vessels, and tumors, providing the robot with a precise, continuously updated procedural map [12]. Furthermore, this spatial awareness is complemented by the ability to precisely track surgical instruments in both position and orientation, a capability foundational for autonomous actions and safety alerts [13]. In addition to identifying anatomy and tools, these systems can also detect and characterize pathological tissues, augmenting the surgeon's diagnostic capabilities [14]. Collectively, these perception skills culminate in the ability to generate high‐fidelity 3D scene reconstructions from 2D video streams, endowing the robot with true spatial awareness. In essence, CV transforms the medical robot from a “blind executor” reliant on external commands into a “perceptive observer” with situational awareness, laying the essential groundwork for all higher‐level autonomous functions [15].

However, situational awareness for autonomy should extend beyond the immediate surgical field. For AI‐powered robots to advance from precision assistance to higher autonomy, perception must cover the larger procedural environment, including the patient's whole body and head pose, team member positions and actions, instrument availability and state, device settings, workspace constraints, and any moving or obstructing objects within the room [16, 17]. Importantly, these elements change continuously during a procedure and may directly affect the safety, timing, and feasibility of subsequent actions. Therefore, the robot must not only “see” anatomy and tools, but also represent the broader scene in a coherent, updatable way that supports real‐time decision‐making.

Equally critical is modeling dynamic interactions and predicting what may happen next [18]. Modern learning‐based perception can be extended from static recognition to temporal understanding by capturing motion patterns and cause–effect relationships among the patient, instruments, and staff [19]. Such predictive capability enables proactive behaviors, such as anticipating patient movement, detecting impending occlusions, forecasting instrument handover or next‐step workflow transitions, and issuing early safety alerts before a hazardous state occurs. In this sense, CV and machine learning provide not only perception, but also environment‐level comprehension and short‐horizon prediction, which are key requirements for progressing from accurate execution to context‐aware autonomy [20].

### 2.2 Deep Reinforcement Learning for Autonomous Decision‐Making and Control

Once a robot can perceive its environment, the subsequent critical challenge is to determine how to act. Deep reinforcement learning (DRL) provides a powerful framework for learning complex control policies, especially in unstructured and unpredictable environments [21]. This end‐to‐end learning approach is an important catalyst for the transition of medical robotics from task automation to genuine cognitive autonomy. Its potential is particularly evident in the automation of repetitive yet skill‐intensive surgical subtasks, such as suturing and knot‐tying, where robots can learn policies that approach or match expert‐level performance in stability and efficiency [22]. For instance, Shahkoo et al. developed a DRL method based on a continuous action space that learns an optimal tissue tensioning strategy for autonomous soft‐tissue cutting, improving precision for complex cutting patterns [23].

Beyond static tasks, the true power of DRL lies in dynamic path planning. This allows an agent to autonomously navigate through deformable anatomical pathways—such as a beating heart or a peristaltic colon—by continuously adapting its path to safely reach a target [24]. To address the challenges in path planning for flexible robotic needles, Lin et al. proposed a DRL method that integrates kinematic and mechanical models. Their framework, which incorporates obstacle avoidance, target attraction, and a retraction mechanism, significantly improves the efficiency and precision of the needle insertion trajectory [25].

Furthermore, DRL facilitates more sophisticated forms of human–robot collaboration when paired with learning‐from‐expert approaches such as inverse reinforcement learning (IRL). Ireddy et al. applied IRL to model real‐world clinical decision data from physicians assessing Type 2 Diabetes Mellitus risk. This approach aims to infer the underlying reward functions and latent preferences behind their decisions, which can then be used to train or constrain DRL policies. Consequently, DRL equips medical robots with the critical ability to learn and adapt, enabling them to make optimal decisions in dynamic clinical scenarios and serving as a key enabling technology for achieving higher levels of robotic autonomy [26].

### 2.3 NLP and LLMs for Human–Robot Interaction

As robotic capabilities become more advanced, the efficiency of the human–robot interface emerges as a new bottleneck. NLP, and particularly the rise of LLMs, is reshaping the paradigm of human–robot interaction, moving it from cumbersome physical controls to seamless, natural‐language dialogue [27]. The integration of LLMs provides a multifaceted communication layer between the surgeon, the robot, and clinical data. The most immediate application is voice‐based control, which allows surgeons to command robotic arms or endoscopic views using simple verbal commands, thereby freeing their hands and cognitive load to focus on the surgical task [28]. Beyond intraoperative commands, LLMs serve as a powerful tool for clinical information synthesis, capable of processing a patient's entire electronic health record preoperatively and providing the surgeon with a concise verbal summary. This capability extends to postoperative workflows, where LLMs can help draft structured surgical reports by organizing key events and instrument usage recorded during a procedure [29]. The scope of LLM‐driven interaction also includes patient‐facing applications, where rehabilitation and service robots can engage in natural conversations to provide coaching, reminders, and companionship. LLMs are effectively dismantling the communication barrier between humans and machines, transforming the robot into an intelligent assistant that can listen, speak, and understand, but clinical deployment requires safeguards for reliability, privacy, and safety.

### 2.4 AI and Digital Twins for Surgical Planning and Simulation

Ensuring the safety and success of a robotic procedure begins long before the first incision. The combination of AI and Digital Twin technology is transforming surgical planning from an “experience‐based art” into a “data‐driven science” [30]. A digital twin is a patient‐specific virtual model of anatomy and physiology, typically built from medical imaging and other clinical data. The synergy between AI and this model creates a powerful ecosystem for preoperative planning, simulation, and intraoperative guidance. This ecosystem begins with personalized surgical rehearsal, where surgeons perform virtual procedures on the patient's digital twin to test different approaches while AI models predict the outcomes, thereby identifying the optimal strategy preoperatively [31]. This virtual environment also serves as an ideal risk‐free sandbox for predicting and refining the robot's behavior, allowing for the optimization of control parameters to prevent potential collisions and ensure smooth execution. It is also the ideal environment for training DRL agents for autonomous tasks [32].

The insights gained from this exhaustive digital planning are then seamlessly translated into the operating room through intraoperative navigation and augmented reality. Here, the preplanned optimal path and critical anatomical structures are overlaid onto the surgeon's view, providing a real‐time “GPS” for the procedure [33]. By enabling exhaustive exploration of possibilities in the digital realm before acting in the physical world, the combination of AI and Digital Twins dramatically enhances the predictability, precision, and safety of robotic surgery, serving as a cornerstone for the vision of personalized medicine. In 2023, Yuk et al. reported what they described as the first totally robotic minimally invasive anterior and posterior (circumferential) lumbar fusion, combining the da Vinci system for anterior lumbar interbody fusion and the Globus ExcelsiusGPS system for posterior pedicle screw placement, demonstrating initial safety and feasibility [34].

## 3 AI‐Driven Revolutions in Clinical Domains

The term “robot” was first introduced by Czech writer Karel Čapek in 1921 [35]. In 1961, the installation of the Unimate robot marked the advent of modern industrial robotics [36]. A pivotal transition from industrial to medical robotics occurred in 1985 when the Unimation Puma 200 performed CT‐guided stereotactic brain biopsies with high precision, demonstrating the potential of robotic systems in surgical interventions and laying the groundwork for the specialized development of medical robots. Subsequent systems such as PROBOT, ROBODOC, Handy1, and MIT‐MANUS further diversified the clinical applications of robotic technology across urology, orthopedics, assistive care, and rehabilitation [37, 38, 39, 40, 41, 42, 43]. By the late 20th century, the introduction of the da Vinci Surgical System heralded a new era in minimally invasive surgery [44, 45, 46, 47]. Through iterative advancements—including the S, Si, Xi, and SP platforms—the system significantly enhanced surgical visualization, dexterity, and precision via multi‐degree‐of‐freedom wristed instruments, 3D vision augmentation, and a master–slave control architecture [48, 49, 50, 51, 52].

**FIGURE 1:**
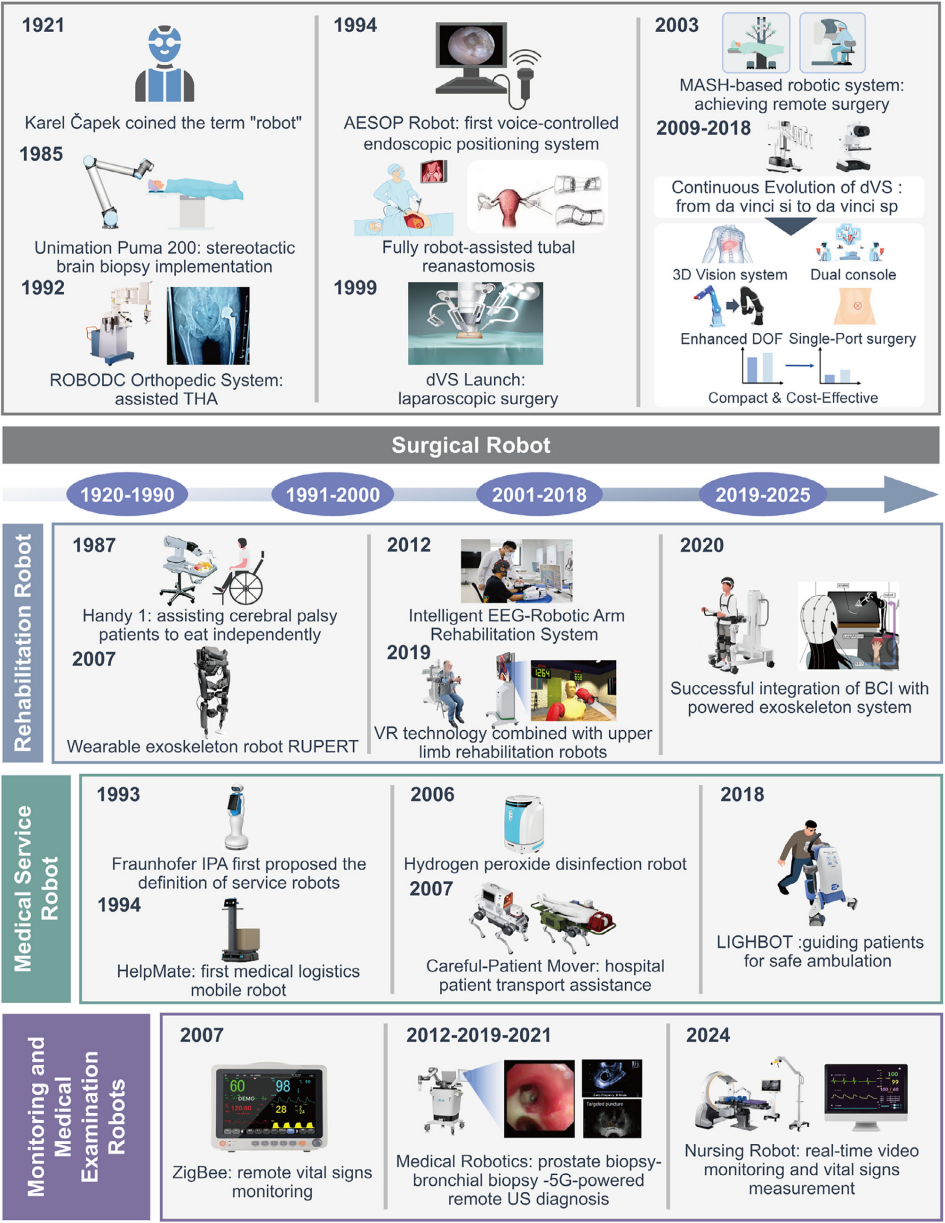
Since Karel Čapek first introduced the concept of “robots” in 1921, medical robotics has evolved into four mature categories by 2024. Medical Service Robots: fraunhofer IPA first defined service robots. The debut of HelpMate, the first medical service robot, spurred the development of intelligent robots for disinfection, patient transport, logistics, and triage services. Surgical Robots: unimation Puma 200 demonstrated the surgical potential of robotics. The da Vinci Surgical System, with its 3D vision, dual consoles, and single‐port technology, has significantly advanced minimally invasive surgery. Furthermore, the MASH system has enabled remote surgical operations. Rehabilitation Robots: handy 1 marked the first generation of rehabilitation robots. Integrating VR, BCI, and exoskeleton technologies, these robots significantly improved therapeutic outcomes. Monitoring & Medical Examination Robots: the ZigBee system laid the foundation for remote monitoring robots. Examination robots have achieved clinical application, effectively performing prostate biopsies, bronchoscopy, and remote ultrasound diagnostics.

Today, driven by AI, robotics is not merely augmenting medical procedures but revolutionizing them. This chapter recategorizes contemporary medical robots based on their transformative impact on clinical domains, focusing on three core areas: intelligent surgery, adaptive rehabilitation, and hospital operational efficiency. We will systematically analyze how AI‐powered robotics is reshaping these fields, as illustrated in Figure 1.

### 3.1 Intelligent Surgical Robotics: From Precision to Autonomy

**FIGURE 2:**
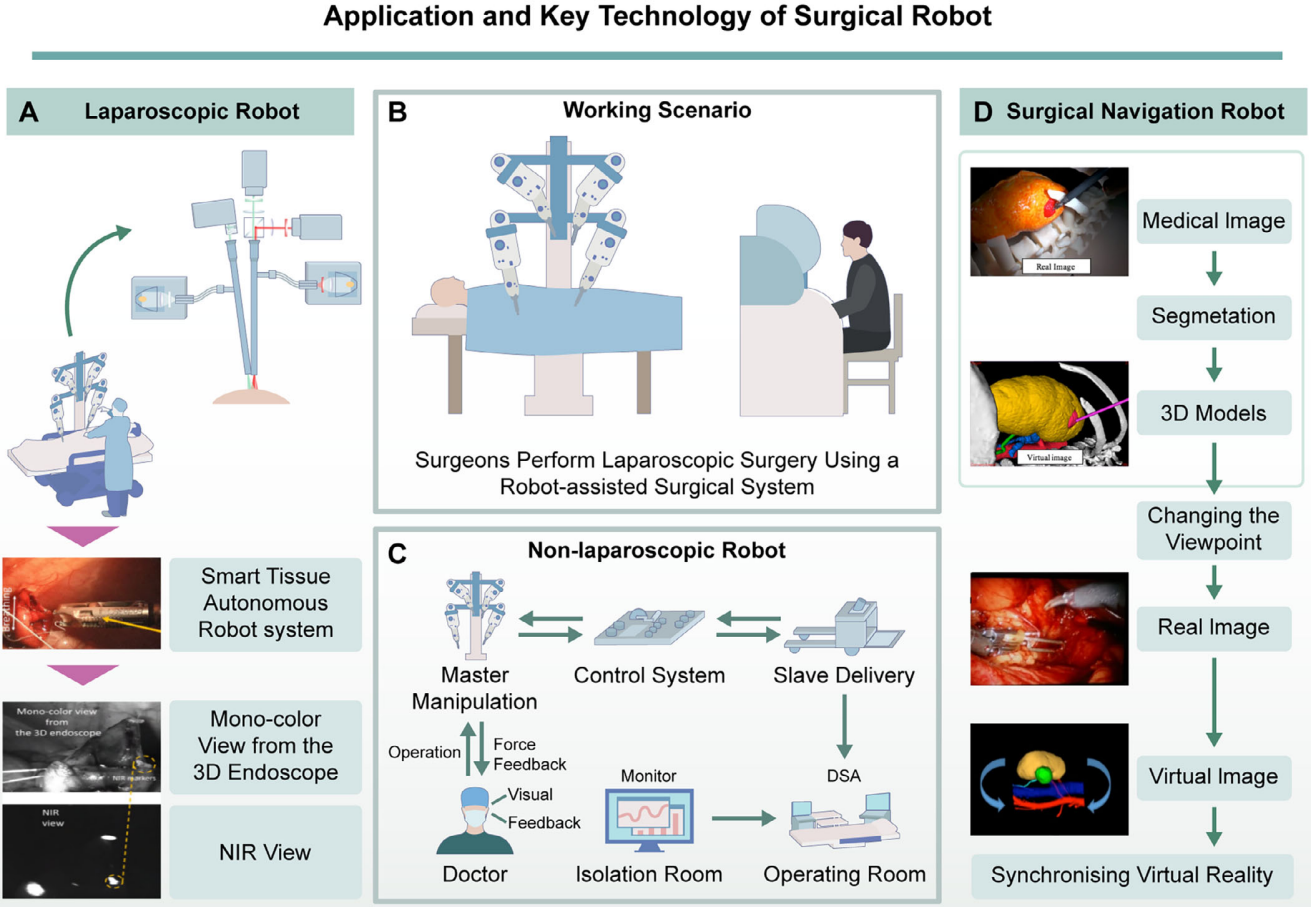
Surgical robotic applications and key technologies. (A) Laparoscopic Surgical Robot: integrates the Smart Tissue Autonomous Robot system with 3D endoscopy and NIR light sources. (B) Operational Scenario: surgeons use a control console to manipulate robotic arms for minimally invasive procedures. (C) Non‐Laparoscopic Surgical Robot: features force feedback, visual feedback, and control systems for remote operation within an isolation chamber, ensuring efficiency and safety. (D) Surgical Navigation Robot: utilizes medical imaging and NIR light sources for image segmentation, 3D modeling, perspective adjustment, and real‐time image registration, enhancing intraoperative decision‐making and surgical precision.

Surgical robots are evolving from teleoperated master–slave systems that enhance precision to intelligent partners capable of semiautonomous or autonomous actions. This evolution is advancing surgical practice to unprecedented levels of personalization and cognitive support. By integrating visual, tactile, and auditory functions, modern surgical robots can automate multiple tasks, effectively reducing the impact of variables such as patient positioning, instrument accuracy, and surgeon experience on procedural outcomes, including precision and safety in conventional procedures [53, 54, 55]. These intelligent systems are becoming core drivers of the next generation of surgical innovation. The journey from precision enhancement to intelligent autonomy is evident across two main categories of surgical robots: operator‐assisted robots and surgical navigation robots, as detailed in Figure 2.

Initially, the focus was on enhancing the surgeon's dexterity and precision. The primary application of the operator‐assisted robots is in laparoscopic surgery; however, they are also used in other surgical specialties, including vascular surgery, orthopedic surgery, and cardiac surgery. Laparoscopic robots address limitations in operational adaptability, sensory feedback, and visualization, which are inherent challenges in traditional laparoscopic surgery [56]. Regarding the enhancement of surgical instrument flexibility, the single port orifice robotic technology (SPORT), EndoMaster, Titan Medical Inc.’s SPORT Surgical System, and the Master–Slave Surgery Robot System enhance the operational flexibility of surgical equipment through advanced mechanical design and multi‐joint technology [57, 58].

In non‐laparoscopic fields, precision has been equally paramount. [59, 60]. Flow‐driven navigation technology, based on fluid dynamics, enables precise catheter control within blood vessels with real‐time feedback. This technology is of great importance in the field of vascular interventional surgery, as it enables the precise execution of surgical procedures [61, 62]. Several systems, including the CorPath GRX, Magellan System, RVIRC, and Pancaldi System, have shown significant potential in improving procedural accuracy and safety [63, 64, 65, 66, 67]. Building on this foundation of precision, surgical navigation robots integrate real‐time imaging and sophisticated algorithms to facilitate preoperative planning and intraoperative guidance, representing a critical step toward procedural automation [68, 69]. For preoperative planning, the Aquabeam and Mazor X Stealth Edition systems utilize 3D imaging to create customized surgical plans [70, 71, 72]. A number of intraoperative navigation systems are currently available, including the KUKA KR3, ARTORG Robotics System, Renishaw neuromate, SEEG MRI navigation systems, OrthAlign, needleless fluoroscopy‐guidance robots, and Tianji Robotics System. Each of these systems is capable of providing accurate positioning and navigation information to support tasks such as instrument alignment and implant placement, reducing manual localization effort. These systems employ different technical approaches to attain intraoperative positioning and navigation.

In short, the “revolution” in surgery is no longer mere mechanical refinement; it is the embedding of perception, reasoning, and shared control into clinical flow, enabling auditable partial autonomy under human oversight.

### 3.2 Adaptive Rehabilitation and Assistive Robotics

Rehabilitation robots are intelligent devices designed to facilitate patients’ functional recovery training, representing a significant advancement in the field of rehabilitation medicine [73]. These devices play a vital role in optimizing rehabilitation outcomes and improving patients’ quality of life. The incorporation of advanced technologies, such as AI, machine learning, sensing technology, and virtual/augmented reality, has significantly advanced the control accuracy, real‐time feedback, and interactive capabilities of rehabilitation robots [74]. This section summarizes the application and development of these technologies in rehabilitation, focusing on musculoskeletal and cognitive rehabilitation robots.

#### 3.2.1 Musculoskeletal Rehabilitation Robot

Musculoskeletal rehabilitation robots are advanced devices that aid the recovery of patients with musculoskeletal impairments by providing individualized rehabilitation programs to restore muscle strength in the arms and legs [75]. These robots play a significant role in modern rehabilitation medicine, with a wide range of applications in rehabilitation training to facilitate the restoration of normal motor function in patients [76, 77, 78]. Furthermore, as technology advances in the field of rehabilitation, neurological rehabilitation is also being increasingly integrated into musculoskeletal rehabilitation. For example, the application of brain–computer interface (BCI) and virtual reality (VR) rehabilitation robots is becoming increasingly sophisticated, offering patients innovative rehabilitation methods.

**FIGURE 3:**
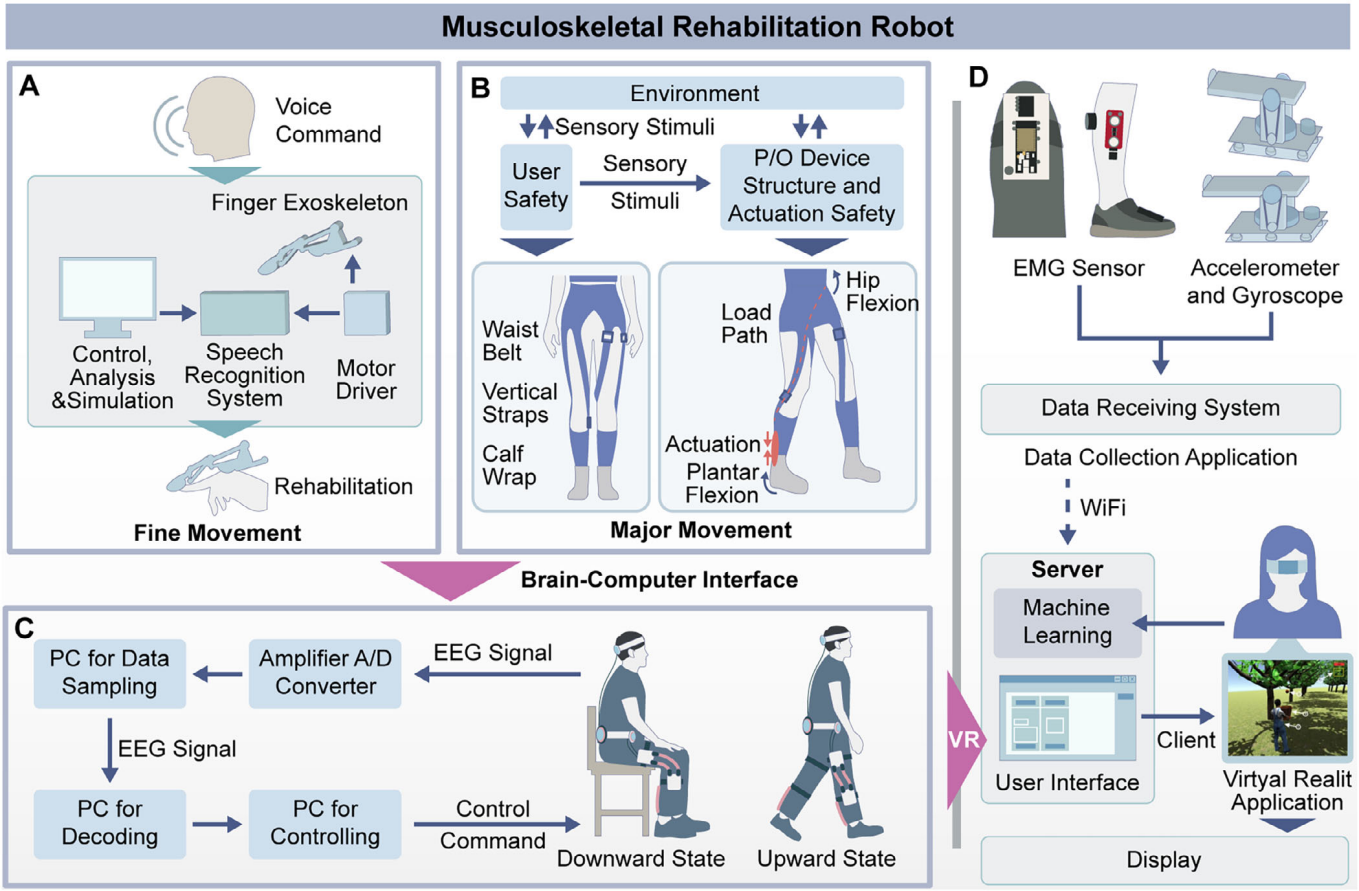
Musculoskeletal rehabilitation robot applications and key technologies. (A) Fine Motor Rehabilitation: integrates voice control, motion analysis, and simulation systems to achieve hand fine motor rehabilitation via a finger exoskeleton device. (B) Gross Motor Rehabilitation System: utilizes a lower‐limb exoskeleton to assist patients in large‐range motion training, with real‐time feedback ensuring safety. (C) BCI Rehabilitation System: acquires and decodes EEG signals to enable motion intention recognition and rehabilitation control. (D) VR‐Enhanced Rehabilitation System: combines EMG sensors, accelerometers, and machine learning algorithms to optimize rehabilitation efficacy through VR‐based training environments.

These can be classified into two categories based on the rehabilitation objectives: those designed for gross motor rehabilitation of large muscle groups and those designed for rehabilitation of small muscle groups. Robots for large muscle groups are used to treat conditions affecting the quadriceps, gluteus maximus, and deltoid muscles in the upper and lower limbs. Their goal is to assist patients in regaining fundamental motor functions, including support, ambulation, pushing, and pulling [79]. The rehabilitation robot designed for small muscle groups is intended to facilitate the restoration of fine motor skills, such as grasping and pinching, which are essential for activities of daily living. These skills are typically exercised by the small muscle groups in the hands and fingers. The applications and key technologies of musculoskeletal rehabilitation robots are shown in Figure 3.

Robots for small muscle groups (e.g., wrist rehabilitation robots, RUPERT system, ARMin system) focus on restoring fine motor skills such as grasping and pinching by improving the flexibility and coordination of hand movements [80, 81, 82, 83, 84, 85, 86]. Gravity‐compensation systems are also a significant area of research, as they reduce the effective load on the limb and help ensure stability and precision during rehabilitation [87].

The integration of BCI and VR technologies has facilitated groundbreaking advancements. BCI technology captures a patient's brain signals to control external devices, which can support motor recovery by linking motor intent to assisted movement, even when neuromuscular pathways are impaired [88, 89, 90]. VR technology constructs three‐dimensional virtual scenarios for rehabilitation training, enhancing sensory feedback and engagement and supporting improvements in muscle strength and joint control accuracy, thereby improving the effectiveness of rehabilitation [91, 92].

#### 3.2.2 Cognitive Rehabilitation Robot

**FIGURE 4:**
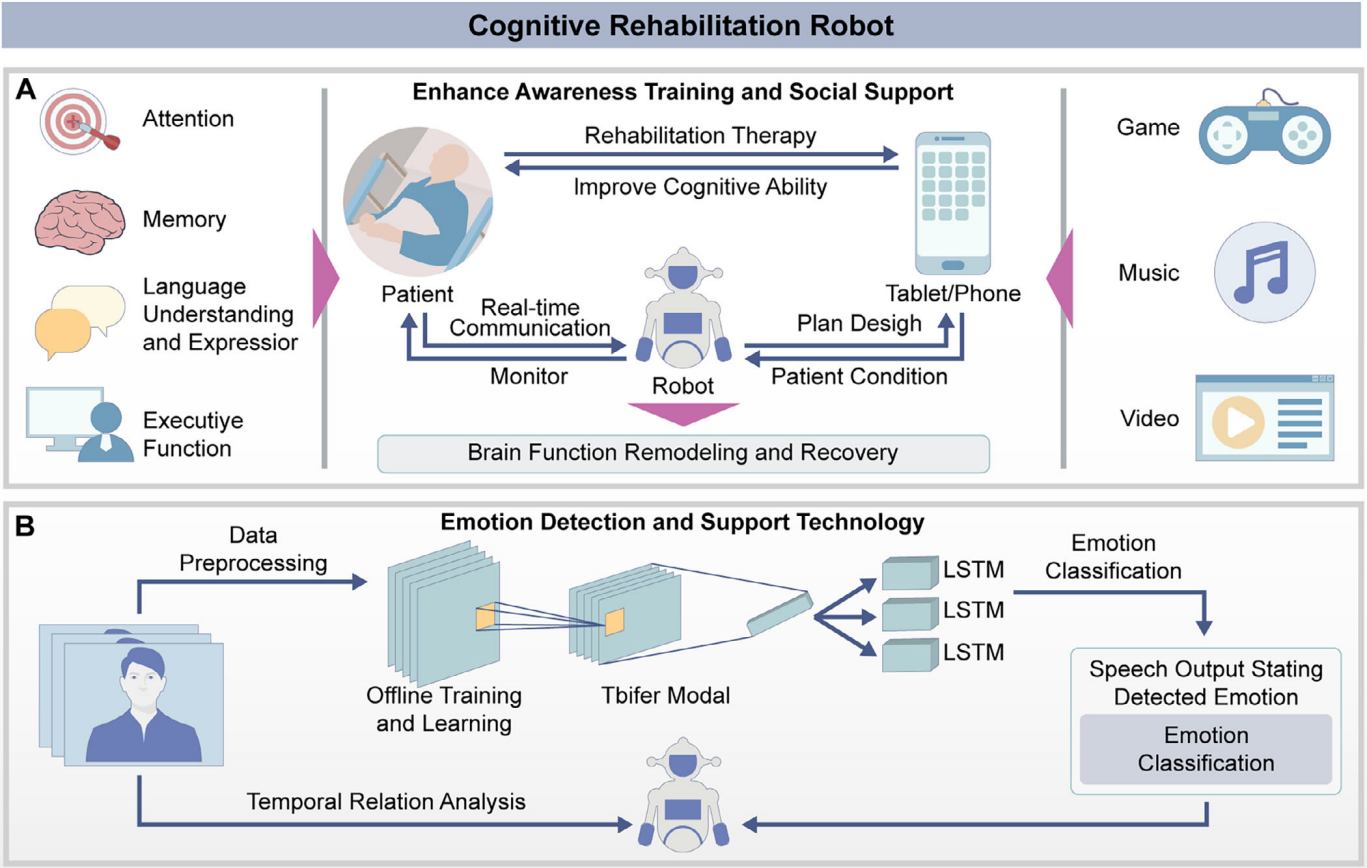
Cognitive rehabilitation robot applications and key technologies. Cognitive rehabilitation robots aim to improve patients’ cognitive abilities such as attention, memory, language comprehension, and executive function (A), and design personalized rehabilitation plans through interaction between the robots and smart devices, as well as real‐time communication and treatment. This class of robots can also support the remodeling and restoration of brain function (B), and promote the reorganization of neural networks and functional recovery.

New technological developments in cognitive rehabilitation mark a significant turning point in neurological rehabilitation. The use of social assistive robots in cognitive training and social interaction has led to rapid development in this field [93, 94, 95]. Cognitive rehabilitation robots can be divided into two categories based on their primary functions and application areas. The first category enhances patients’ cognitive abilities and social interactions. These robots are primarily utilized to assist individuals diagnosed with Parkinson's disease, stroke, and dementia [96, 97, 98]. Through structured cognitive exercises and guided interactions, they aim to improve memory, attention, and social skills. The second category of robots is designed to detect and support emotions through multimodal analysis and interactive technology. This technology improves the social responses and emotional experiences of children with autism [99, 100]. The applications and key technologies of cognitive rehabilitation robots are shown in Figure 4.

Emotion recognition and supportive technologies encompass empathetic interaction, keyword recognition, and music therapy. Non‐humanoid robots, dialogue robots, and robot‐based music therapy platforms facilitate one‐on‐one interactions with users, influencing their emotions and promoting psychological rehabilitation. In 2013, Fernando developed the Social Robot Maggie, which integrates voice and facial expression analysis functions for emotion detection and adjusts dialogue strategies to enhance user satisfaction [101]. In 2021, Erel et al. utilized empathic gestures from non‐humanoid robots, modeled after psychotherapists, to enhance emotional support for participants [102]. In 2022, Huang et al. developed a dialogue robot that identifies stressors by extracting keywords and provides emotional support by combining psychological traits to help cope with difficulties [103]. In the same year, Feng et al. proposed a novel robot‐based music therapy platform that improved accuracy in motor control tasks and supported better performance in following instructions and displaying appropriate behaviors in music game interactions through an autonomous social interaction system and music intervention [104]. These robots exhibit distinct characteristics; Maggie focuses on emotion detection and adaptive dialogue strategies; the non‐humanoid robot improves emotional support through empathetic gestures; the dialogue robot delivers targeted emotional support by integrating psychological traits; and the robot music therapy platform enhances behavioral and social responses, particularly in children with autism spectrum disorders.

### 3.3 Transforming Hospital Workflow and Operational Efficiency

Beyond direct patient intervention and rehabilitation, AI‐driven robots are revolutionizing the operational backbone of healthcare facilities. By automating routine, labor‐intensive, and critical support tasks, these systems enhance efficiency, improve safety, and free up human staff to focus on high‐value patient care. This transformation is evident across several key areas, including surveillance and monitoring, disinfection, logistics, and patient triage and guidance, thereby streamlining hospital workflow.

#### 3.3.1 Surveillance and Monitoring Robots

In the medical field, surveillance robots have significantly enhanced patient observation and real‐time operational efficiency through sensing technology and automation [105]. These robots support efficient navigation, monitoring, medication delivery, and home care [106, 107]. For example, MEDROVER delivers navigation control and real‐time AI‐driven fall detection via a mobile app, integrates ultrasonic sensors with mapping algorithms to generate a 2D environmental map for precise navigation, and incorporates a speaker system to enable human‐like communication with users [108]. The iWARD system enhances activity tracking in healthcare settings by remotely measuring body temperature, heart rate, electrocardiogram, respiratory rate, and body acceleration through sensors, while issuing alerts during critical patient conditions [109]. MedRob is used for automatic medication distribution and vital‐sign measurement, improving medication management efficiency via RFID and IoT [110]. Mobile home care robots can monitor a comprehensive range of vital signs while providing mobility support for remote patient–clinician interactions [111].

#### 3.3.2 Disinfection Robots

Disinfection robots have significantly improved environmental hygiene and safety in hospitals [112]. These robots use innovative technology to provide efficient and automated disinfection, reducing manpower requirements and improving operational accuracy and reliability [112, 113]. Disinfection robots, such as the UVD Robot, LightStriker, Tru‐D SmartUVC, and AIMBOT, employ UV‐C light, chemical sprays, or atomization, and even combine multiple modes to achieve thorough and efficient disinfection. UVD robots, for instance, autonomously navigate hospital wards and operating rooms, using UV‐C light to inactivate microorganisms and, under specified conditions, achieve up to 99.99% reduction within approximately 10 min [114, 115, 116]. LightStriker and Tru‐D SmartUVC provide similar UV disinfection, but due to the line‐of‐sight limitation of UV (especially in shadowed or occluded areas), some shaded areas may not be thoroughly disinfected [117]. AIMBOT covers large areas by spraying or atomizing highly oxidizing disinfectants to compensate for the shortcomings of UV light, but it can also cause corrosion and secondary contamination problems [118, 119]. The difference is that the UVD robot focuses on autonomous movement and UV‐C light disinfection, while the LightStriker and Tru‐D SmartUVC focus on providing automated UV disinfection services, and the AIMBOT covers more areas by spraying disinfectant.

In contrast, Shanghai Taimi Robotics’ TRD‐03 intelligent disinfection robot integrates three disinfection modes: ultraviolet light, ultra‐dry mist hydrogen peroxide, and air filtration to achieve 360° all‐around disinfection and reduce disinfection blind spots [120]. These robots identify high‐risk areas in hospitals and adjust disinfection strategies for refined, quantitative management [121, 122]. Through intelligent and automated means, disinfection robots have strengthened the ability to control and prevent infectious diseases and have played a key role in ensuring public health and safety.

#### 3.3.3 Logistics and Transfer Robots

Transfer robots are automated systems deployed in medical environments to move patients, medical equipment, pharmaceuticals, and other items, streamlining logistics and enhancing patient care [123, 124, 125, 126, 127, 128]. In terms of intelligent transport of goods, the TUG robot can autonomously navigate, avoid obstacles, and even interface with elevators wirelessly to efficiently transport trays, medications, and medical records [129]. HOSPI and the CIoT robot enhance the efficiency of delivering medicines and specimens using advanced navigation and IoT technology [130, 131]. In the context of patient transfer, C‐Pam employs a pioneering vertical stacking conveyor belt configuration. This innovation facilitates the vertical and horizontal movement of patients, thereby minimizing the risk of secondary injury and reducing the workload of medical staff [132, 133]; E‐Pat significantly improves transfer comfort by adjusting the contact angle between the patient and the conveyor belt and optimizing the inclined plane design [134]. Advanced systems like the Baize robot and RoNA use environmental perception and human‐body recognition to ensure safe and comfortable patient transfers, with some capable of lifting up to 500 pounds [135, 136].

#### 3.3.4 Triage and Guidance Robots

The development of triage and guidance robots has significantly enhanced outpatient service efficiency and elevated the patient experience. Intelligent patient services are an important concept for innovative hospitals that use information technology, including medical service robots, to provide more convenient, faster, and more accurate medical services [137].

Triage robots, such as Pepper, Temi, Navii, and Sanbot, demonstrate remarkable advantages over manual services by leveraging integrated AI, multimodal interaction, real‐time data analysis, and automatic obstacle avoidance technologies. These robots quickly respond to patient needs and deliver personalized services, significantly improving the efficiency of outpatient services and the overall patient experience. The Pepper robot has been reported to improve service satisfaction after being deployed in Belgian hospitals, supported by its data processing capabilities and emotion‐recognition functions [138, 139, 140]. Multimodal SoR detects user emotions by recognizing faces, body language, and speech to improve personalized service levels and patient experience [141, 142, 143]. Viguro Robot uses AI and sensors for real‐time data analysis and navigation to improve hospital facility efficiency and service quality [144]. LIG HBOT is designed for the visually impaired and elderly. It uses a touch screen to navigate to a destination, and the speed depends on the force exerted by the user, which improves the user experience [145].

Furthermore, research has validated these benefits. Ma et al. found that a mobile intelligent guide robot significantly improved the efficiency of location consultation, process guidance, specialty clinic recommendation, and health education, reducing outpatient workload and improving patient experience [146]. The robot developed by Siao et al. guides users to the correct location by voice, has translation capabilities, and can provide emergency contact information, while detecting obstacles and reducing hazards [147]. Triage robots in hospitals play an important role in disseminating information about the hospital and guiding patients and visitors. They can tirelessly receive large numbers of visitors and direct them to the doctor. For children, robots can improve the medical experience by providing a pleasant experience to reduce their sense of discomfort [148]. In addition, some systems emphasize vital‐sign monitoring and interactive support, depending on the deployment setting and clinical goal.

## 4 Critical Challenges and Ethical Considerations

**FIGURE 5:**
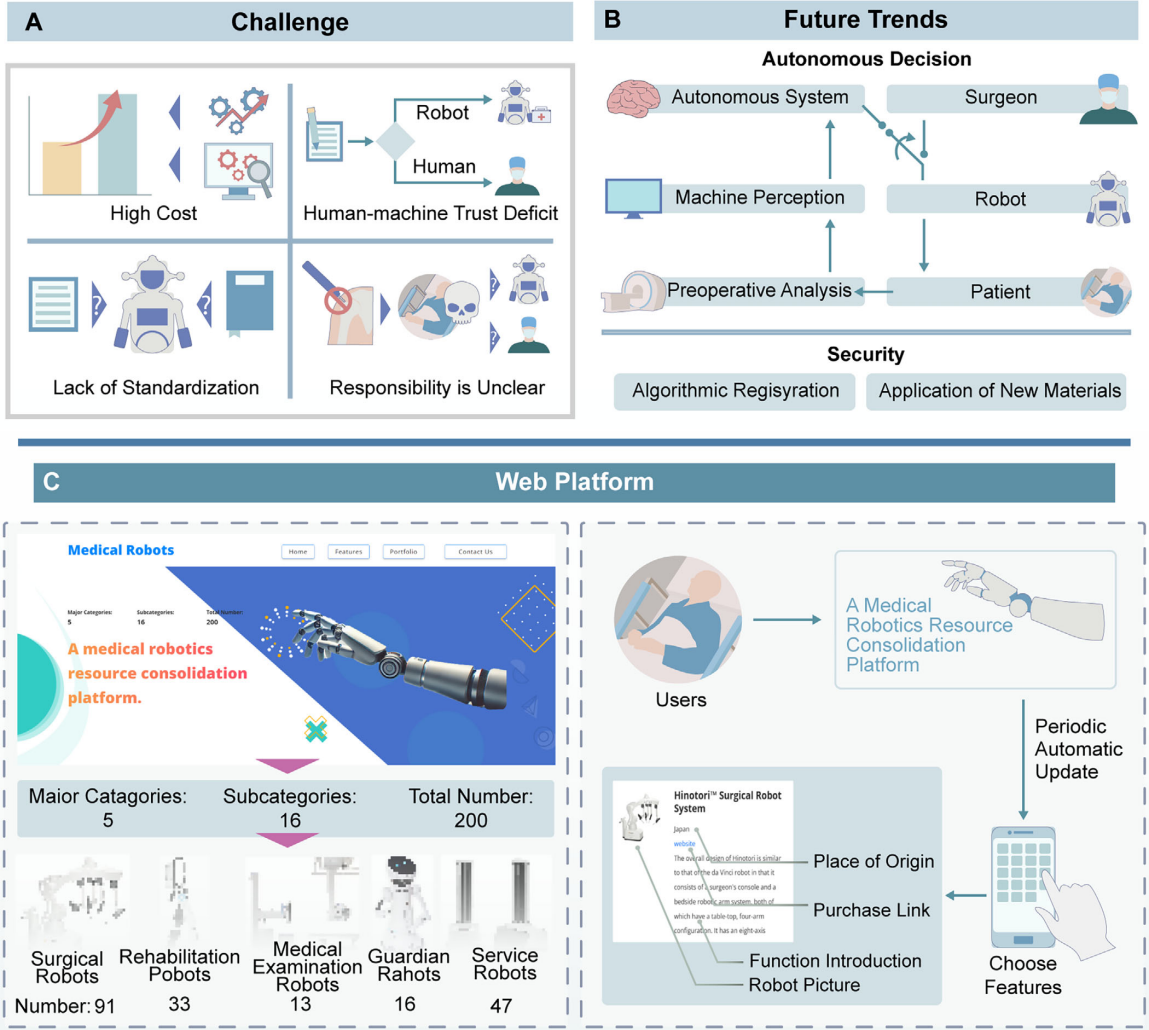
Medical Robotics: Challenges, Trends & Resource Platform. (A) Current Limitations: high costs, suboptimal human–robot collaboration, low standardization, and ambiguous liability mechanisms. (B) Future Trends: focus on autonomous systems via enhanced physician‐robot coordination, perception optimization, patient engagement, preoperative analytics, AI/material integration, improving safety and reliability. (C) Resource Platform: a self‐developed hub for medical robot classification, enabling information search, functional selection, and real‐time medical tech updates.

Despite the increasing use of medical robotics in clinical practice, substantial challenges persist, impeding their advancement and broader adoption. This section provides a thorough examination of the significant limitations confronting medical robots in clinical applications (Figure 5A). These challenges span technical hurdles, safety and verification dilemmas, ethical and legal frameworks, and practical barriers to clinical translation.

### 4.1 Technical Hurdles: Data Scarcity, Model Generalizability, and Real‐Time Performance

The performance of AI‐driven robotics is fundamentally dependent on the quality and quantity of data used for training. A primary technical barrier is data scarcity and data quality variability. High‐quality, annotated medical data can be difficult to obtain due to privacy concerns, the cost of expert labeling, and inherent data imbalances. This scarcity directly impacts model generalizability—the ability of an AI system to perform accurately on new, unseen data from different patient populations, clinical settings, or different devices and workflows.

These data limitations manifest in suboptimal real‐time performance and reliability. For example, in HCI, robots may not fully understand or respond to human intent, which can affect the accuracy, safety, and user experience in surgical workflows. These challenges are mainly reflected in several aspects. First, the limitations of existing technology in high‐precision surgery lead to error accumulation during surgery. Existing HCI robot control technology mainly uses two methods: Impedance Control and Admittance Control [149, 150]. In impedance control, the system adjusts the output force based on the input displacement. However, in compliant and variable environment, this control method is difficult to achieve high‐precision operation. Admittance control often fails to correct operator errors or hand tremors in a timely manner, and may even amplify these errors, thereby affecting surgical precision and user experience [151].

These challenges in real‐time performance are also critical in teleoperated robot operations. The inherent communication delays and data loss in telemedicine robot operations are particularly critical for remote surgery over long distances. Most remote operation systems encounter some time delay in practical applications, which poses a serious challenge to the synchronization of command exchange and operational accuracy, ultimately affecting the stability and efficiency of the system [152].

### 4.2 The Safety and Verification Dilemma: Ensuring Reliability of “Black Box” AI Systems

The integration of advanced computational models, particularly those based on deep learning, into medical robotics introduces a profound verification and validation dilemma that represents a paradigm shift from conventional software engineering [153]. Unlike procedural, deterministic software that is amenable to exhaustive validation against predefined specifications, the stochastic and data‐driven nature of these AI systems limits formal verification across the full spectrum of potential clinical scenarios. This is especially true for their response to rare “edge cases” or out‐of‐distribution data. Consequently, such systems often function as opaque or “black box” entities; they may demonstrate high fidelity in training and testing environments but are susceptible to exhibiting unexpected and often inscrutable failure modes in clinical practice, which has profound implications for patient safety [154].

This deficit in verifiability directly engenders a crisis of clinical trust and operational reliability. For instance, phenomena such as operational latency or deviation from an operator's intended trajectory are not merely HCI issues; they are symptomatic of a more fundamental deficit in predictability and interpretability. In a high‐stakes surgical intervention, when a robotic system exhibits unexpected behavior, the opacity of its underlying model prevents the clinician from discerning the cause of the action. It becomes impossible to distinguish between a sensor artifact, a software anomaly, or a deliberative, AI‐driven safety maneuver based on the system's interpretation of the surgical field. This causal ambiguity critically erodes operator confidence and undermines the fluid human–robot collaboration requisite for complex medical procedures [155].

Mitigating this dilemma necessitates a new validation paradigm that moves beyond conventional performance metrics toward a holistic framework encompassing robustness, transparency, and interpretability. Promising avenues of research include explainable AI, which develops techniques to render model decisions transparent and provide human‐interpretable rationales, such as highlighting influential anatomical features in radiological images for clinical review [156]. Concurrently, the application of formal methods to provide guarantees that a model's output will remain within pre‐specified safety boundaries, complemented by adversarial testing to systematically probe for vulnerabilities, is crucial for assessing resilience against unforeseen data variations [157]. Ultimately, assuring the safety of AI‐driven medical robotics transcends mere regulatory compliance; it necessitates the establishment of a robust foundation of verifiable trust among clinicians, patients, and the autonomous systems integral to the future of healthcare delivery.

### 4.3 Ethical and Legal Imperatives in AI‐Driven Medicine

The integration of AI into medical robotics fundamentally reconfigures the landscape of medical responsibility, shifting from a clear doctor–patient dynamic to a complex network involving clinicians, patients, hardware manufacturers, software developers, and network infrastructure providers. This transformation erodes traditional models of accountability, creating urgent ethical and legal challenges that require robust frameworks to ensure patient safety and trust [158].

A primary challenge is the attribution of liability. In the event of an adverse outcome caused by an autonomous or semiautonomous system, the lines of responsibility become unclear [159]. Is the surgeon liable for trusting the AI's recommendation? Is the hospital responsible for inadequate training or maintenance? Does liability lie with the manufacturer for a hardware malfunction, or with the AI developer for a flawed algorithm? This creates a complex “liability chain” that traditional malpractice law, centered on human negligence, is ill‐equipped to handle [160]. The difficulty in pinpointing whether an error stemmed from flawed code, biased training data, a hardware defect, or improper clinical use presents a significant obstacle to legal resolution and could slow adoption if not addressed proactively.

Closely linked to accountability are the imperatives of transparency and fairness. Many advanced AI models, particularly in deep learning, operate as “black boxes,” making it difficult to trace their decision‐making process [161]. This opacity obstructs not only post‐incident legal investigations but also the cultivation of clinical trust, as physicians may be hesitant to rely on systems whose reasoning they cannot understand or audit [162]. Furthermore, algorithmic bias presents a significant ethical risk [163]. AI systems trained on unrepresentative datasets may perpetuate or even amplify existing health disparities, leading to discriminatory or suboptimal outcomes for certain patient populations. For instance, a diagnostic algorithm trained predominantly on data from one demographic might be less accurate for others, raising serious questions about equity and justice in healthcare delivery [164].

Addressing these issues requires more than just adapting existing laws. It necessitates the development of new, interdisciplinary frameworks cocreated by ethicists, legal experts, engineers, clinicians, and policymakers. These frameworks must establish clear guidelines for data governance, algorithmic validation, continuous monitoring, and transparent reporting to ensure that the deployment of AI in medicine enhances patient care while upholding core principles of justice, fairness, and accountability over the full system lifecycle [165].

### 4.4 Clinical Translation Barriers: Regulatory Pathways and Human–Robot Trust

Beyond theoretical and technical advances, significant practical barriers hinder the widespread clinical translation of medical robotics. These include navigating complex regulatory pathways, overcoming economic and training hurdles, and fundamentally, fostering durable human–robot trust.

A primary barrier to the clinical translation and global adoption of intelligent medical robots is the fragmented landscape of regulatory pathways and the absence of harmonized technical standards. While regional authorities establish market access requirements—such as FDA premarket clearance in the United States and CE marking under the medical device regulation (MDR) in Europe—these frameworks tend to emphasize procedural compliance rather than technical uniformity across borders [166, 167]. This inconsistency complicates international market entry and creates uncertainty for developers.

This regulatory fragmentation is compounded by a critical gap in technical standards tailored to AI‐driven systems. Foundational standards like ISO 13485 for quality management and IEC 62304 for the software lifecycle were not designed for the complexities of adaptive, nondeterministic models [168, 169]. More specific standards, like IEC 80601‐2‐77 for robotically assisted surgical equipment, primarily address hardware reliability rather than the emergent behaviors of the integrated AI [170]. However, the standards landscape is beginning to evolve to address these complexities. For instance, the international AI committee ISO/IEC JTC 1/SC 42 is developing foundational standards, including ISO/IEC 23894 for AI risk management [171]. In parallel, guidance documents are emerging to bridge the gap, such as AAMI TIR34971, which interprets the application of risk management (ISO 14971) specifically to AI and machine learning [171]. Despite these nascent efforts, the absence of a fully harmonized and globally adopted framework for AI model validation, performance benchmarking, and post‐market surveillance undermines interoperability and slows technical progress by perpetuating uncertainty for developers.

Economic factors also present substantial hurdles. The development of medical robots faces multiple cost challenges across the entire pipeline, from research and development to commercialization. R&D involves substantial investments, high technological complexity, and the need for repeated testing and verification under stringent regulatory and performance constraints. Quality control and certification requirements make it expensive to source custom components, and the devices themselves are expensive. Take the da Vinci surgical robot as an example. Its cost typically ranges from $1.5 to $2.5 million, plus annual maintenance costs and significant per‐procedure expenses, all of which can increase overall surgical expenditures, and these costs are often ultimately borne by the patient [148, 172]. The cost of ownership should also not be ignored. The additional cost of using the da Vinci surgical robot is approximately $1600–$3200 per procedure. The unit price of its instruments and accessories is usually between $1800 and $4600, and they can only be used for about 10 times, which further increases the financial burden on medical institutions [173, 174].

Additionally, significant limitations exist in clinician training and skill acquisition. Due to the complexity and high technical requirements of robotic surgical systems, surgeons require significant time and resources to master these skills. Yet, the limited availability and high cost of existing training platforms hinder effective learning, leading to a shortage of proficient robotic surgeons and extended training periods. Studies have shown that most residency programs do not formally incorporate training in robot‐assisted surgical skills, and the high cost is a major barrier [175].

Finally, the success of clinical adoption hinges on establishing human–robot trust. Robots’ limited capacity to accommodate individual patient preferences and preserve interpersonal interaction can weaken patients’ confidence and create a psychological distance between doctors and patients. From the patient perspective, it has been reported that robots may erode patients’ sense of security by failing to address specific individual needs and reducing interpersonal interaction [176]. Although 82.9% of Colombian healthcare professionals have a positive attitude toward robotics, there is a general concern in the medical community that robots may replace human labor, and there is a lack of in‐depth understanding of how robots are applied in clinical settings [177]. The study by McDermott et al. shows that participants have deep‐seated concerns about the acceptance of new technologies, believing that they may create alienation and distance between surgeons and patients [178].

## 5 Future Perspectives and Converging Frontiers

Medical robots have already demonstrated significant benefits in surgical assistance, rehabilitation support, diagnostic monitoring, and medical services, improving accuracy and efficiency. However, limitations in automation and safety, along with issues such as high costs, operational complexity, lack of standardization, and system latency, have hindered their widespread use. To achieve broader clinical acceptance, a paradigm shift is required, moving beyond incremental improvements toward fundamental breakthroughs. The following sections explore pivotal future perspectives, focusing on the converging frontiers of AI, robotics, and materials science that will define the next generation of medical robots (Figure 5B).

### 5.1 The Road to Full Autonomy: Key Breakthroughs Needed

Autonomous decision‐making refers to a robot's ability to perceive and analyze environmental information and, based on that analysis, independently select and execute optimal action plans to complete predetermined tasks with minimal or no human intervention [179]. Autonomous decision‐making is a key indicator of a robot's intelligence, enabling it to adaptively respond to complex and dynamic environments [180, 181]. However, the path to safe and reliable full autonomy is fraught with challenges, necessitating breakthroughs in several key areas.

Enhancing environmental perception and data fusion is fundamental. High‐precision sensor systems form the foundation of a medical robot's environmental perception capabilities [182]. By integrating sensors such as cameras, tactile and force sensors, and acoustic detectors, these robots can gather multidimensional data, including 3D surgical images, tissue‐tool interaction forces, and environmental conditions like temperature and humidity. This data provides the robot with rich environmental information, enabling it to make more accurate decisions and perform actions [183, 184]. The next frontier lies in real‐time, reliable fusion of this multimodal data to create a comprehensive and dynamic understanding of the surgical field, potentially surpassing human perceptual limits.

Beyond perception, achieving robust algorithmic performance in unpredictable environments is a major hurdle. Current systems still face challenges such as delayed decisions, incorrect execution, and an inability to handle unexpected situations [185]. These shortcomings can manifest as delayed decision‐making, incorrect execution, or inability to respond to unexpected situations [186, 187, 188]. A critical area requiring a breakthrough is the robustness of core algorithms, such as registration and navigation. While current technologies excel in static environments, they falter when target tissues exhibit unpredictable motion and deformation [189, 190]. The complexity of surgical scenarios and the lack of large‐scale, standardized dynamic datasets for training limit the generalization capabilities of current AI models [191, 192]. To overcome this, future research must focus on developing deformation‐aware models and comprehensive datasets that encompass organ deformation and instrument‐tissue interactions, enabling algorithms to maintain high‐precision operations in complex surgical environments [193].

To address these challenges, researchers are exploring advanced multimodal models to enhance the accuracy and reliability of task planning and execution. For instance, algorithms leveraging deep learning and reinforcement learning, such as Deep Q‐Networks and Deep RL models, have been applied to create an intelligent surgical navigation system. This system enables automatic target identification and real‐time decision‐making in complex surgical scenarios [194]. Meanwhile, autonomous decision‐making systems integrated with LLMs, such as multimodal GPT‐4V, aim to enhance embodied task planning by combining natural language instructions with robotic visual perception, offering forward‐looking insights into bridging the gap between human–machine interaction with the environment [194].

Furthermore, true autonomy requires not only intelligent algorithms but also mechanically robust and adaptive hardware. This is where advances in materials science become indispensable for enhancing safety and performance. A major challenge lies in developing materials that combine high flexibility, conductivity, and biocompatibility [195]. Future material design should prioritize fatigue resistance, self‐healing capabilities, and environmental adaptability [196]. For instance, self‐healing polymers and 4D‐printed smart materials, which can dynamically respond to stimuli like body temperature, promise to create more intelligent, durable, and responsive medical robots [197, 198]. The synergy between advanced algorithms and intelligent materials is crucial for creating next‐generation autonomous systems that are not only smart but also safe, reliable, and perfectly adapted to the human body [199].

### 5.2 Embodied AI and Human‐in‐the‐Loop Collaborative Systems

Beyond pure autonomy, the future lies in creating intelligent systems that learn through direct physical interaction and collaborate seamlessly with human experts. Embodied AI represents this paradigm shift, where robots develop intelligence not just from preexisting datasets but also through active engagement with their physical environment [200]. In medicine, this means a robot could learn the nuances of tissue manipulation by physically performing tasks and receiving feedback, leading to more adaptive and intuitive behaviors [201].

However, in high‐stakes medical scenarios, full autonomy may not always be desirable. A Human‐in‐the‐Loop collaborative system offers a compelling alternative, blending AI's precision and data‐processing power with the surgeon's judgment and ethical oversight [202]. In this model, the AI acts as a copilot, not an autopilot. It can suggest optimal incisions, highlight critical structures in real‐time, or automate repetitive and cognitively demanding subtasks, while the surgeon retains ultimate control, able to intervene or override the AI at any moment [203]. This collaborative approach, often termed shared autonomy, enhances safety, builds trust, and leverages the complementary strengths of both human and machine, paving the way for systems that are both highly intelligent and responsibly deployed in clinical practice.

### 5.3 The Fusion of AI, Nanorobotics, and Synthetic Biology for Targeted Therapy

One of the most profound future frontiers is the convergence of AI with nanorobotics and synthetic biology, promising to revolutionize medicine at the cellular level. Nanorobots, miniaturized devices operating at the nanometer scale, are being designed to navigate the bloodstream to deliver drugs directly to cancer cells, perform highly localized microsurgical operations on individual cells, or monitor specific biomarkers of disease [204]. The primary challenge for these devices is autonomous navigation and decision‐making within the complex, dynamic environment of the human body. This is where AI is critical. AI algorithms can be used to control the propulsion of nanobots, process sensory data to identify target cells, and trigger the release of therapeutic payloads selectively when specific pathological conditions are met [205].

This frontier is further expanded by synthetic biology, which involves reengineering biological organisms such as bacteria or immune cells to function as “living” robots. These bio‐robots can be programmed to seek out tumors and produce anticancer agents in situ, or to repair damaged tissue [206]. The fusion of AI with these biological systems could enable multilayered decision‐making logic, allowing them to make complex therapeutic decisions based on multiple biological inputs [207]. While significant hurdles in biocompatibility, control, and in vivo tracking remain, this convergence points toward a future of ultra‐precise, personalized medicine delivered by intelligent, microscopic agents.

### 5.4 Swarm Robotics for In Vivo Diagnosis and Cooperative Intervention

Moving beyond individual robots, the concept of swarm robotics—deploying a large number of simple, cooperative robots—holds immense potential for in vivo applications. Inspired by social insects, a robotic swarm can perform tasks that would be impossible for a single, larger robot, offering enhanced robustness, scalability, and parallelism [208]. In a medical context, a swarm of microrobots could be ingested or injected to collaboratively map a region of the gastrointestinal tract, form a temporary scaffold to help heal a wound, or collectively deliver a therapeutic agent with high spatial precision [209].

The control of such swarms relies heavily on decentralized AI. Each robot in the swarm operates based on simple, local rules and communicates with its immediate neighbors, leading to complex, emergent collective behavior. AI is essential for designing these control policies, enabling the swarm to adapt its overall shape, navigate intricate biological channels, and coordinate its actions without a central controller [210]. For example, AI algorithms could guide a swarm to aggregate at a bleeding site to form a clot or to collectively generate localized heat through controlled hyperthermia to destroy tumor cells. Overcoming challenges in real‐time tracking and controlling these swarms in vivo is a key research focus, but the potential for minimally invasive, cooperative interventions is a powerful driver for future innovation.

### 5.5 The AI‐Augmented Surgeon: The Future of Human–Machine Synergy in Medicine

Ultimately, the future of medical robotics may not be the replacement of the human surgeon, but rather the creation of the AI‐augmented surgeon. This vision focuses on human–machine synergy, where technology enhances and expands the surgeon's natural abilities in perception, cognition, and action [211].

In this paradigm, AI will serve as an ever‐present intelligent assistant. For perception, augmented reality overlays, powered by AI image analysis, will project patient‐specific 3D models of tumors, blood vessels, and nerves directly onto the surgeon's view of the patient, making the invisible visible [212]. For cognition, AI will analyze data from large‐scale surgical datasets encompassing thousands of prior procedures to provide real‐time decision support, predicting potential complications, suggesting optimal tool paths, and dynamically personalizing the surgical plan mid‐procedure [213]. For action, robotic platforms will continue to filter out hand tremors and scale movements for microsurgery, but with an added layer of AI‐driven safety, creating “virtual no‐fly zones” around delicate structures. This synergy will not only improve the precision and safety of procedures but also has the potential to democratize surgical expertise, allowing more surgeons to perform at the level of the world's best. The operating room of the future will be an integrated ecosystem where the surgeon's skill is amplified by intelligent robotics, ushering in a new era of surgical excellence.

To address the challenge of fragmented information in the field, we have engineered an integrated resource hub for medical robotics developers (available at https://medicalrobotics.shuaiailab.cn/). The platform serves a dual purpose: first, it provides a consolidated overview of commercially available robotic systems, with direct links to official websites (Table 1). Second, it aims to accelerate the research and development lifecycle by providing a structured purchasing guide for critical equipment parts. This guide features curated links to official brand suppliers, accompanied by detailed product descriptions and visual aids to simplify component selection and procurement (Figure 5C).

**TABLE 1:** Representative commercialized medical robots and their technological characteristics.

International Standard	Brands	Products	Clinical Application	Core Technology	Key Features/Innovation	Official Website Link
Surgical robots	Intuitive Surgical	Zeus	Minimally invasive and multi‐organ surgery	Master–slave telemanipulation, 3D endoscopic visualization	One of the earliest tele‐surgical systems; precursor to da Vinci platform	https://www.intuitive.com/en‐us
	Medicaroid	Hinotori Surgical Robot System	Urologic and gynecologic surgery	Multi‐joint robotic arms, ergonomic console design, 3D imaging	First domestically developed Japanese surgical robot emphasizing compactness and precision	https://www.medicaroid.com/en/
	CMR Surgical	Versius surgical robot	General laparoscopic surgery	Modular arm design, haptic feedback, AI‐assisted camera control	Compact modular design; supports surgeon‐specific ergonomic setup	https://cmrsurgical.com/
	Asensus Surgical	Senhance Surgical System	Minimally invasive surgery	Eye‐tracking camera control, force feedback, digital laparoscopy	Integrates machine vision for real‐time instrument tracking	https://www.asensus.com/
	avateramedical	Avatera	Pelvic and abdominal surgery	Fully reusable instruments, 3D HD vision, ergonomic interface	Cost‐effective, sterilizable components reduce per‐procedure costs	https://avatera.eu/home
Rehabilitation robots	Barrett Technology	Burt	Upper‐limb neuromuscular rehabilitation	Torque‐controlled actuators, impedance control	Provides natural backdrivability for fine motor recovery	https://barrett.com/
	hpcosmos	Robowalk	Gait and lower‐limb rehabilitation	Robotic treadmill with adaptive resistance	Adjustable gait assistance with real‐time feedback	https://www.hpcosmos.com/
	Aldebaran Robotics	NAO	Cognitive and social rehabilitation	NLP, emotion recognition, interactive AI	Enhances motivation and engagement during therapy	https://aldebaran.com/
	AIST	PARO	Dementia and cognitive therapy	Artificial emotional intelligence, tactile sensors	Robotic seal with emotional response for neuropsychological rehabilitation	https://www.aist.go.jp/
	humanware	Motore	Upper‐limb rehabilitation	Force sensors, motion capture, AI training adaptation	Provides adaptive training intensity based on user feedback	https://www.humanware.com/en‐international/
Medical examination robots	GI View	AER‐O‐SCOPE	Colonoscopy and gastrointestinal imaging	Disposable colonoscope, embedded camera navigation	Capsule‐based imaging robot for safer colonoscopy	https://www.giview.com/
	Cassling	CorPath GRX	Endovascular intervention	Robotic catheter control, haptic interface	Enables remote percutaneous coronary intervention (PCI)	https://www.cassling.com/
	Universal Robots	UR5e	Medical manipulation platform	Collaborative robot arm, force–torque sensing	Used in ultrasound, biopsy, and lab automation	https://www.universal‐robots.com/
	Olympus	ENF‐P4 Endoscope System	ENT endoscopy	High‐resolution flexible optics, narrow‐band imaging	Compact design for improved visualization of upper airway	https://www.olympus‐vn.com/
	Landwind Medical	Radiography system Apollo Pro	Radiographic imaging	Digital radiography, AI‐based image optimization	Integrates AI post‐processing for exposure correction	https://www.landwindmedical.com/
Service robots	Verve Motion	Vemotion	Exosuit for nursing and logistics support	Soft‐robotic actuators, wearable sensors	Reduces musculoskeletal load for healthcare workers	https://vervemotion.com/
	vecnarobotics	Vecna BEAR	Patient lifting and transport	Force‐assisted lift mechanism, AI navigation	Reduces caregiver injury risk during transfers	https://www.vecnarobotics.com/
	ronna	RONNA	Neurosurgical assistance	Image‐guided stereotaxy, robotic arm precision	Enables high‐accuracy cranial trajectory planning	https://www.ronna‐medical.hr/
	Xenex	Xenex LightStrike	Hospital disinfection	Pulsed‐xenon UV disinfection, autonomous mobility	Kills > 99.9% pathogens in < 10 min	https://xenex.com/
	robotnik	I‐SUPPORT	Patient mobility and hygiene assistance	Sensor fusion, compliant control	Provides semiautonomous care assistance for elderly	https://robotnik.eu/
Assistive robots	Tru‐D SmartUVC	Tru‐D	Hospital disinfection	UV‐C dose monitoring, autonomous navigation	Validated UV‐C disinfection with automatic exposure mapping	https://tru‐d.com/
	Blue Ocean Robotics	UVD	Surface and air disinfection	UV‐C light, SLAM navigation	Autonomous 360°udisinfection with safety sensors	https://www.blue‐ocean‐robotics.com/
	Karcher	RoboCoV Cleaner	Hospital and laboratory disinfection	Spray atomization, autonomous navigation	Combines chemical misting and path optimization	https://www.karcher.cn/cn/
	Dreame	mini UVC	Mobile UV sterilizer	Compact UV‐C sterilization, IoT connectivity	Lightweight sterilization robot for small environments	https://mall.dreame.tech/
	Blue Ocean Robotics	Blue Ocean Robotics	Multipurpose robotic service base	Modular robot platform, AI navigation	Provides scalable base system for hospital‐specific customization	https://www.blue‐ocean‐robotics.com/

## 6 Conclusion

This review delineates a transformative paradigm shift within medical robotics, driven by the deep and systematic integration of AI. Our analysis confirms that the field has progressed beyond developing mere high‐precision tools to creating intelligent partners capable of perception, reasoning, and adaptation. In surgical robotics, AI‐powered CV is not just enhancing accuracy but is fundamentally redefining the cognitive load on surgeons, allowing them to focus on high‐level strategy rather than low‐level execution. Similarly, in rehabilitation and diagnostics, the shift is from static, one‐size‐fits‐all protocols to adaptive, patient‐centric systems that learn and evolve with an individual's unique physiological data. This evolution from automated instrument to cognitive collaborator represents the central finding of our review: AI is endowing medical robots with a nascent but functionally meaningful form of clinical intelligence, paving the way for a future of cooperative, data‐driven healthcare.

Despite these remarkable advances, the path from laboratory innovation to widespread clinical adoption is obstructed by a triad of interrelated challenges that demand deeper analysis. Firstly, the socio‐economic barrier of high cost and complex maintenance is not merely a financial issue; it raises critical questions of accessibility and equity in healthcare. Secondly, the technical‐trust barrier stems from the inherent complexity of AI‐integrated systems. Issues like submillimeter motion control and the “black box” nature of some deep learning models create a deficit in clinical trust, which is further compounded by a lack of standardized validation protocols for ensuring algorithmic safety and reliability. Finally, the human‐factor barrier, reflected in inadequate operator training and suboptimal human–robot interaction, reveals that technology is outpacing our capacity to integrate it seamlessly into established clinical workflows. These challenges are not independent; they form a systemic resistance to translational adoption that must be addressed holistically.

To dismantle these barriers, future research must pursue a multipronged strategy centered on advancing autonomy, intelligence, and physical embodiment. A primary objective should be the development of robust and explainable AI. This involves moving beyond current models to more sophisticated architectures, such as reinforcement learning for dynamic path planning in unpredictable surgical environments, and leveraging multimodal sensor fusion (e.g., combining vision, force, and tactile data) to create a more comprehensive “understanding” of the clinical scene. Furthermore, the physical form of robots must coevolve with their intelligence. The integration of intelligent materials, such as shape‐memory polymers and self‐healing composites, is not a minor upgrade but a crucial step toward creating more durable, biocompatible, and intrinsically safer devices that can better adapt to the human body. These technological pursuits are not merely incremental improvements; they represent strategic imperatives to build the next generation of trustworthy and effective medical robots.

Ultimately, realizing this technological vision is contingent upon fostering a deeply integrated and institutionalized collaboration among engineers, AI scientists, and clinicians. This requirement transcends a simple call for teamwork; it necessitates the creation of a unified innovation ecosystem. In this ecosystem, clinical needs, articulated by physicians, directly inform the algorithmic development by AI scientists and the hardware design by engineers. Conversely, technological possibilities must be transparently communicated to clinicians, allowing them to cocreate applications and validation standards from the earliest stages of development. Formalized frameworks such as joint research labs, interdisciplinary training programs, and shared clinical innovation platforms are essential infrastructures for this coevolution. Only through such structural integration of cross‐disciplinary expertise can we ensure that the development of AI‐driven medical robotics remains grounded in clinical reality, ethically sound, and sharply focused on the ultimate goal: delivering a new era of personalized, precise, and profoundly more effective patient care.

## Author Contributions

J.S., G.Y., Q.X., and Y.L. conceived the conceptualization and designed the paper. F.C., H.C., T.Y., and R.W. wrote the paper. Y.W., X.Z., J.L., K.L., D.H., X.B., Z.M., D.Y., and Z.W. participated in discussion. All authors have read and approved the final manuscript.

## Funding

This study is supported by the Ministry of Science and Technology of the People's Republic of China (grant no. 2021ZD0201900), the National Natural Science Foundation of China (grant no. U24A2014, 12090052, 12375334, 12274356), and the Natural Science Foundation of Ningbo (grant no. 2024Z148), and the Wenzhou Science and Technology Bureau's Project ZY2024002

## Ethics Statement

The authors have nothing to report.

## Conflicts of Interest

The authors declare no conflicts of interest.

## Data Availability

The authors have nothing to report.
